# The effect of ursodeoxycholic acid in dissolving gallstones formed after laparoscopic sleeve gastrectomy: retrospective cohort study

**DOI:** 10.1007/s00423-025-03656-1

**Published:** 2025-03-07

**Authors:** Fatih Buyuker, Medeni Sermet, Mehmet Sait Ozsoy, Salih Tosun, Özgür Ekinci, Hakan Baysal, Orhan Alimoglu

**Affiliations:** Istanbul Medeniyet University Faculty of Medicine Goztepe Prof. Suleyman Yalcin City Hospital, Istanbul, Turkey

**Keywords:** Laparoscopic sleeve gastrectomy, Gallstones, Gallbladder, Obesity surgery, Dissolving, Ursodeoxycholic acid

## Abstract

**Purpose:**

Rapid weight loss that often occurs after laparoscopic sleeve gastrectomy (LSG) has been linked to an increased risk of gallstone formation. This study aimed to investigate whether ursodeoxycholic acid could be an effective alternative treatment for gallstone dissolution, potentially offering a nonsurgical option for patients requiring gallstone removal.

**Methods:**

This retrospective study analyzed 88 patients who underwent LSG and subsequently developed gallstones between 2017 and 2023. Fifty-one patients who received UDCA treatment were compared to 37 patients who did not receive UDCA. Demographic and clinical characteristics and gallstone dissolution rates were analyzed using SPSS v25.0.

**Results:**

Gallstones dissolved in 60% of patients who received UDCA treatment, and symptoms such as dyspepsia decreased. A stone diameter of less than 5 mm was associated with a higher treatment success rate. The number of hospitalizations and admissions due to gallstone symptoms has decreased. The side effects were mild and did not require treatment discontinuation.

**Conclusions:**

UDCA treatment is an effective option for the resolution of gallstones after LSG. However, surgery may be more appropriate for treating larger stones. The results of this study suggest that UDCA is an effective intervention for reducing gallstone-related complications following LSG.

## Introduction

Laparoscopic sleeve gastrectomy (LSG) is a widely performed bariatric surgery that effectively treats obesity. However, rapid weight loss after LSG is a significant risk factor for the development of gallstone disease (cholelithiasis). This rapid weight reduction leads to an increased cholesterol concentration in the bile and decreased gallbladder motility, both of which contribute to gallstone formation. Additionally, deficiencies in bile salts and disturbances in bile flow exacerbate the process. Alterations in fat metabolism due to weight loss further impact bile composition and promote gallstone formation [1, 2]. While gallstone prevalence is 10–15% in the general population, this rate is approximately double in patients undergoing bariatric surgery [3–5]. Factors, such as age, sex, obesity severity, and genetic predisposition, also increase this risk.

Although surgery is the primary treatment for symptomatic gallstones, additional surgical interventions can increase the risk of complications in patients undergoing bariatric procedures. Some patients may avoid surgery because of concerns over potential complications, lengthy recovery, financial burdens, alternative treatments, and a lack of information. Pharmacological treatment options are valuable in such cases. Ursodeoxycholic acid (UDCA) is one of the most commonly used agents to prevent gallstone formation after bariatric surgery. UDCA enhances cholesterol solubility in bile, potentially shrinking or dissolving gallstones and reducing the need for surgical intervention [6, 7]. While gallstone formation is often asymptomatic, complications such as biliary colic, cholecystitis, pancreatitis, and choledocholithiasis can arise, adversely affecting the patients’ quality of life. Thus, a key postoperative goal for patients who develop gallstones after LSG is to dissolve the existing stones as an alternative to cholecystectomy. UDCA helps prevent gallstone formation and improves bile flow by modifying the bile composition and reducing hydrophobicity. However, the efficacy and safety of UDCA, particularly in the context of gallstones developing after bariatric surgery such as LSG, remain inadequately explored. Therefore, our study aimed to investigate the efficacy of UDCA treatment and its impact on complications (e.g., dyspepsia, biliary colic, cholecystitis, and choledocholithiasis) in patients with gallstone formation after LSG.

## Materials and methods

This retrospective study aimed to evaluate the efficacy of ursodeoxycholic acid (UDCA) treatment in patients who developed gallstones after laparoscopic sleeve gastrectomy (LSG) at a tertiary care teaching and research hospital in Istanbul between 2017 and 2023. This study was performed in line with the principles of the Declaration of Helsinki. Approval was granted by the Ethics Committee of University Istanbul Medeniyet (Date 11.12.2024/No. 2024-GOSEK-018).

### Patient selection and preoperative evaluation

Routine preoperative evaluation for patients undergoing LSG includes comprehensive laboratory tests such as complete blood count, liver function tests, lipid profile, fasting blood sugar and HbA1c. In addition, upper gastrointestinal endoscopy was performed to detect diseases such as malignancy, ulcer, hiatal hernia and abdominal ultrasonography (US) was performed to evaluate the presence of gallbladder stones or sludge in all patients. Patients with gallbladder stones or sludge detected before surgery were evaluated for their symptoms and symptomatic and asymptomatic (relative indication) simultaneous cholecystectomy was recommended. However, patients who did not accept surgery were followed up without surgical intervention in the postoperative period. US was performed between 1 and 30 days before surgery. Patients with preoperative stone detection were excluded from the study.

### The inclusion criteria were as follows

-Presence of cholesterol-containing gallstones in the gallbladder confirmed by ultrasound imaging in patients who underwent LSG surgery.
-Adherence to a prescribed follow-up regimen three months after surgery.
-Routine ultrasonography to identify cholesterol gallstones.
-The patient requested an alternative treatment for cholecystectomy.
-Completion of at least six months of UDCA therapy.
-For patients receiving UDCA treatment, the minimum treatment period was 12 months from the start of the therapy.
-Age of at 18 years.

### The exclusion criteria were as follows

-Presence of bile pigment stones.
- Presence of preoperative gallstones or sludge.
- History of cholecystectomy.

Liver failure or other conditions contraindicated UDCA treatment.

Out of 403 patients, 88 met the inclusion criteria. These patients were monitored for at least 12 months following post-LSG gallstone detection and were divided into two groups: one group of 51 patients received UDCA treatment, while the other group of 37 patients did not receive UDCA treatment.

### Postoperative follow-up and imaging protocol

In the postoperative period, patients underwent routine clinical and laboratory evaluations at 1, 3, 6, 9 and 12 months after surgery. Abdominal ultrasonography was also performed postoperatively to evaluate gallstone formation and stone characteristics. After the 12th month, additional ultrasonography imaging was performed only in cases of biliary tract symptoms such as biliary colic or dyspepsia.

### UDCA treatment protocol

UDCA is not routinely prescribed after LSG. Instead, it was applied selectively based on patient preference and clinical indications. UDCA treatment was recommended to patients diagnosed with postoperative gallstones but without acute complications. UDCA was prescribed at a dose of 750 mg in the evening and continued for at least 6 months. The decision to terminate the treatment was made based on follow-up ultrasonography findings and improvement of symptoms.

### Data collection and analysis process

In this study, patient demographic information (age, sex, and body mass index), comorbid conditions (such as diabetes, hypertension, and obstructive sleep apnea syndrome), gallstone formation times, stone sizes and compositions identified via ultrasonography, treatment duration, laboratory results, and post-treatment follow-up processes were retrospectively collected from the hospital information management system. The occurrence of gallstone-related complications, including dyspepsia, biliary colic, cholecystitis, and choledocholithiasis, as well as the implementation of ERCP interventions. The presence of cholesterol stones was corroborated by meticulous stone analysis, and the response times to UDCA therapy and stone dissolution rates were determined. All patients in the UDCA group received a single dose of 750 mg of ursodeoxycholic acid in the evening.

### Demographic and clinical characteristics of patients

A statistical analysis of the demographic and clinical characteristics of the 88 patients included in the study revealed that the mean age was 38.7 ± 12.6 years in the UDCA treatment group and 40.2 ± 10.9 years in the non-treatment group. The proportion of female patients was higher than that of male patients in both the groups (76.4% in the UDCA group and 72.9% in the non-UDCA group). No significant differences were observed between the groups with regard to body mass index (BMI), diabetes, hypertension, or other comorbidities. The overall mean BMI was 31.5 ± 4.5 kg/m². The mean diameter of gallstones, as confirmed by ultrasonography, was 6.3 ± 2.1 mm across all patients (Table 1).

**Table 1:** Demographic and clinical characteristics of the participants receiving and not receiving UDCA treatment

Parameters	UDCA Users (*n* = 51)	Non-UDCA Users (*n* = 37)
Male	12 (23.6%)	10 (28.1%)
Female	39 (76.4%)	27 (72.9%)
Mean Age (Years)	38.7 (19–63)	40.2 (21–64)
Mean BMI (kg/m²)	31.5 (28.3–54.6)	30.8 (26.3–55.8)
Diabetes Mellitus (DM)	10 (19.6%)	8 (21.6%)
Hypertension (HT)	14 (27.5%)	12 (32.4%)
Obstructive Sleep Apnea (OSAS)	6 (11.8%)	4 (10.8%)
Cardiac Diseases	4 (7.8%)	3 (8.1%)
Other Comorbidities	8 (15.7%)	6 (16.2%)
Stone Formation Duration (Months)	9.6	10.3
Stones < 5 mm	30 (58.8%)	23 (62.2%)
Stones > 5 mm	21 (41.2%)	14 (37.8%)

Data are presented as mean (range) or n (%). Comparisons were made using the Chi-square test (*p* < 0.05)

### Statistical analysis

Data were analyzed using the SPSS v25.0 statistical software package. Continuous variables were presented as mean ± standard deviation (SD), while categorical variables were presented as percentages (%). Comparisons between two groups were conducted using the independent samples t-test and the Mann-Whitney U test for continuous variables and the chi-square test for categorical variables. The efficacy of UDCA therapy was evaluated using multivariate logistic regression analysis, considering stone size and composition. The level of statistical significance was set at *P* < 0.05.

## Results

In patients who underwent laparoscopic sleeve gastrectomy (LSG) and developed gallstones, complete dissolution of gallstones was observed in 60% of those treated with ursodeoxycholic acid (UDCA), with an average dissolution time of 4.2 ± 1.5 months. A significant association was found between stone size and treatment success; the dissolution rate was 60.0% for stones smaller than 5 mm compared to 57.2% for stones larger than 5 mm (*p* < 0.05) (Table 2).

**Table 2:** Changes in clinical outcomes over time

Parameters	Baseline UDCA Users (*n* = 51)	6-Month UDCA Users	12-Month UDCA Users	Baseline Non-UDCA Users (*n* = 37)	6-Month Non-UDCA Users	12-Month Non-UDCA Users	*p*-value
Dyspepsia	38 (74.5%)	26 (50.9%)	15 (29.4%)	29 (78.3%)	22 (59.4%)	24 (64.8%)	0.014*
Biliary Colic	27 (52.9%)	16 (31.3%)	14 (27.4%)	19 (51.3%)	20 (54.0%)	23 (62.1%)	0.737
Cholecystitis	1	4 (7.8%)	2 (3.9%)	0	6 (16.2%)	8 (21.6%)	0.625
Choledocholithiasis	0	2 (3.9%)	1 (1.9%)	0	4 (10.8%)	5 (13.5%)	1
ERCP Procedures	0	3 (5.9%)	1 (1.9%)	0	5 (13.5%)	7 (18.9%)	0.563
Hospital Visits	0	32 (62.7%)	21 (41.7%)	0	26 (70.2%)	31 (83.8%)	0.041*
Hospital Admissions	1	6	3	0	10	14	0.032*
Average Visits per Patient	0	1.7	1.2	0	2.2	2.7	0.014*
Cholecystectomy	0	5 (9.8%)	8 (15.7%)	0	9 (24.3%)	12 (32.4%)	1
Stone Dissolution	0	27 (52.9%)	32 (62.7%)	0	0	0	0.001*
Stone Absence	0	22 (43.1%)	29 (56.8%)	0	0	1 (0.3%)	0.001*

*Chi-square test was used for categorical variables, p-value < 0.05. UDCA: Ursodeoxycholic Acid; ERCP: Endoscopic Retrograde Cholangiopancreatography

Dyspepsia symptoms significantly decreased in patients receiving UDCA (from 50.9 to 29.4%, *p* = 0.0041), whereas no such reduction was observed in those not receiving treatment. The incidence of biliary colic remained low in the UDCA group (31.3% at baseline to 27.4%), whereas an increase was observed in those without UDCA therapy (*p* = 0.002). The incidence of cholecystitis was also significantly lower in the UDCA group (*P* = 0.043).

A statistically significant difference was observed between the UDCA and control groups in various clinical parameters related to bile duct stones and their associated complications. Dyspepsia rates at 6 months were 50.9% in the UDCA group and 59.4% in the control group; this difference was not statistically significant (*p* = 0.517). However, at 12 months, dyspepsia rates were 29.4% in the UDCA group and 64.8% in the control group, a statistically significant difference (*p* = 0.001).

Regarding biliary colic, the UDCA group demonstrated lower rates at both six months (31.3% vs. 54.0%, *p* = 0.047) and 12 months (27.4% vs. 62.1%, *p* = 0.002) compared to controls. Similarly, the incidence of other biliary complications, including choledocholithiasis, cholecystitis, and ERCP interventions, was significantly lower in the UDCA-treated group. Specifically, the incidence of cholecystitis was lower in the UDCA group (*P* = 0.042).

The observed difference in choledocholithiasis rates between the two groups was not statistically significant (*p* = 0.106). However, the incidence of complications necessitating endoscopic retrograde cholangiopancreatography (ERCP) and hospital admission was lower in the UDCA group (*p* = 0.03 and *p* = 0.001, respectively). Patients who did not receive UDCA therapy had a higher incidence of antibiotic-requiring conditions and ERCP procedures. At the six-month mark, the proportion of patients requiring ERCP in the UDCA cohort was 5.9% compared to 13.5% in the non-UDCA group. Similarly, the rate of antibiotic-requiring conditions was 18.9% and 7.8% in the non-UDCA and UDCA groups, respectively.

Notable outcomes were observed in the patients who received UDCA, particularly in terms of stone size reduction and dissolution. At the six-month mark, 62.7% of the patients in the UDCA cohort exhibited dissolution of gallstones, whereas no dissolution was observed in the non-UDCA group. After 12 months, complete dissolution or disappearance of stones was recorded in 56.8% of patients in the UDCA cohort (*p* < 0.05) compared to only 0.3% in the non-UDCA group. Additionally, notable discrepancies were observed between the UDCA and non-UDCA groups with respect to the frequency of hospital admissions and number of hospitalizations. During the six-month follow-up period, the mean number of hospital admissions was 1.7 in the UDCA group and 2.2 in the non-UDCA group were recorded. At 12 months, the mean number of admissions was 1.2 in the UDCA group and 2.7 in the non-UDCA group, respectively. Furthermore, a higher hospitalization rate was observed in the non-UDCA group.

No differences were observed in the liver function test parameters between the UDCA and non-UDCA groups at either the six- or twelve-month mark.

### Adverse effects and treatment tolerance

During the course of the treatment, some patients experienced mild adverse gastrointestinal effects. However, when assessed for dyspeptic symptoms, these did not necessitate treatment discontinuation was not necessary. No serious adverse effects or complications were reported and UDCA treatment was generally well tolerated Table 3.

**Table 3:** Changes in biochemical parameters over time

Parameters	Baseline UDCA Users	6-Month UDCA Users	12-Month UDCA Users	Baseline Non-UDCA Users	6-Month Non-UDCA Users	12-Month Non-UDCA Users	*p*-value
AST (U/L)	43 ± 9	45 ± 12	40 ± 10	48 ± 14	50 ± 15	52 ± 14	0.732
ALT (U/L)	47 ± 13	50 ± 15	42 ± 13	53 ± 14	55 ± 17	58 ± 16	0.841
ALP (U/L)	121 ± 27	120 ± 30	110 ± 28	127 ± 20	130 ± 35	132 ± 34	0.874
GGT (U/L)	92 ± 24	80 ± 20	70 ± 18	84 ± 18	85 ± 22	88 ± 21	0.798
Bilirubin (mg/dL)	1.1 ± 0.2	1.2 ± 0.3	1.1 ± 0.3	1.2 ± 0.3	1.4 ± 0.4	1.5 ± 0.5	1
Albumin (g/dL)	3.9 ± 0.2	4.0 ± 0.2	4.1 ± 0.2	4.0 ± 0.1	3.9 ± 0.3	3.8 ± 0.4	1
INR	1.0 ± 0.1	1.1 ± 0.1	1.0 ± 0.1	1.1 ± 0.1	1.2 ± 0.1	1.3 ± 0.1	1

*Two-sample t-test (used for comparing means between groups), p-value < 0.05

AST: Aspartate Aminotransferase, ALT: Alanine Aminotransferase, ALP: Alkaline Phosphatase, GGT: Gamma-Glutamyl Transferase, INR: International Normalized Ratio

## Discussion

This study highlights the effectiveness of UDCA therapy for dissolving cholesterol gallstones in patients who developed gallstones after LSG. UDCA has emerged as an effective treatment option for dissolving cholesterol gallstones in patients unsuitable for or refusing surgery. Notably, therapy significantly reduced the number of hospital visits, admissions, and gallstone-related complications. Mild gastrointestinal side effects were observed during the treatment; however, these symptoms did not hinder continued therapy, supporting UDCA as a safe treatment option.

The incidence of gallstones following LSG ranges from 25 to 45% [8, 9], with rapid weight loss being a major contributing factor to stone formation. Additionally, the necessity of prophylactic cholecystectomy in these patients remains a topic of debate, with some studies suggesting that UDCA may prevent stone formation [10, 11].

In our study, the incidence of gallstone formation was 22.4% over a 24-month follow-up period, with an average time to stone formation of 9.8 months. The observed stone formation rate was consistent with that reported in the literature. No patient received prophylactic ursodeoxycholic acid (UDCA) treatment following surgery. The lack of prophylactic UDCA treatment may have contributed to the high rate of stone formation, given the prophylactic potential of UDCA. The rapid weight loss that frequently occurs following LSG was identified as a significant contributing factor in the development of cholesterol gallstones. As a result of the routine follow-ups, the stones were primarily identified as being in the millimetric range or as biliary sludge, rather than as large stones. The commencement of UDCA treatment prior to the substantial growth of stones and while their hardness remains limited has been associated with a significant dissolution of stones. UDCA has been demonstrated to reduce the risk of bile duct obstruction by promoting the dissolution of cholesterol gallstones. The dissolution of existing stones and the prevention of new ones forming also contribute to a reduction in biliary colic episodes. A single study has indicated that UDCA may have the capacity to dissolve cholesterol stones by reducing cholesterol concentration in bile and lowering the risk of precipitation [12]. Another study recommended surgical intervention for large and pigment stones, as UDCA was found to be ineffective for such types [13]. The diminished dissolution rates observed for larger stones have been attributed to the restricted penetration of bile acids into the stone as its surface area increases [13, 14]. Our findings align with these results and reinforce the prevailing view that UDCA is more efficacious in treating smaller stones. Additionally, the literature indicates that UDCA therapy necessitates prolonged treatment, typically between six and 12 months, for optimal stone dissolution [15]. However, the extended duration of treatment may negatively impact patient compliance.

UDCA has been demonstrated to improve bile acid metabolism, thereby enhancing bile flow and potentially reducing dyspeptic symptoms and biliary colic attacks. Portincasa et al.l. have demonstrated that UDCA has the potential to dissolve cholesterol stones and improve gallbladder function [16]. However, alterations in the biliary system subsequent to weight loss can also impact dyspeptic symptoms. UDCA, being a more hydrophilic bile acid, replaces toxic and hydrophobic bile acids, thereby rendering the bile acid pool safer [17]. Consequently, UDCA reduces inflammation in the gallbladder and bile ducts, resulting in a notable reduction in dyspeptic complaints.

Some studies have indicated that UDCA may have the potential to reduce biliary complications [18, 19]. However, UDCA does not directly prevent complications such as acute cholecystitis, choledocholithiasis, or pancreatitis. Consequently, hospitalization is inevitable when these complications arise. Therefore, the effect of UDCA treatment on reducing hospital admissions related to these complications is limited. There is, however, no clear consensus on whether UDCA directly affects antibiotic usage [20].

The impact of UDCA therapy on the reduction of acute cholecystitis episodes is constrained. Some studies indicate that UDCA may potentially reduce stone formation; however, it may not be an effective treatment for cholecystitis attacks caused by pre-existing stones [21]. In cases of acute cholecystitis, surgical intervention is typically the recommended course of action. UDCA facilitates an increase in bile flow, thereby promoting a more regular emptying of the gallbladder. This can prevent stagnation in the bile ducts, thereby reducing the risk of biliary colic and cholecystitis. In contrast to the literature, our study has demonstrated a reduction in hospital admissions and complication rates. We believe that the key reason for this is the close monitoring of patients, the administration of symptomatic interventions based on the presence of symptoms, and the early treatment associated with the precipitation of cholesterol concentration in gallstones before deepening of the condition.

The impact of UDCA on choledocholithiasis and the necessity for ERCP is constrained. UDCA has been demonstrated to facilitate the dissolution of cholesterol stones by enhancing their solubility within the bile. Nevertheless, the literature indicates that this dissolution effect is typically inadequate for the treatment of choledocholithiasis [22]. UDCA may facilitate an increase in bile flow and the prevention of stagnation within the bile ducts, which could provide long-term benefits in the reduction of new stone formation. Nevertheless, in patients with preexisting choledocholithiasis, this effect is inadequate to obviate the necessity for ERCP.

UDCA may be recommended prophylactically following ERCP to prevent the formation of new stones in the bile ducts; however, it does not reduce the need for ERCP in the presence of choledocholithiasis. Sugiyama et al. have demonstrated that UDCA can reduce the necessity for endoscopic interventions in select patients [23, 24]. Nevertheless, for large and pigment stones, the requirement for ERCP persists.

It has been observed that UDCA does not result in a direct reduction in hospital admissions among patients with cholelithiasis. The impact of UDCA on gallstone dissolution appears to be constrained under specific circumstances [25]. Although it has been proposed that hospital admissions may decline indirectly as a result of the alleviation of gallstone symptoms, UDCA does not exert a direct reducing effect on symptomatic or acute complications. Consequently, its overall impact on hospital admission frequency is minimal [26]. In our patient cohort, however, those receiving UDCA demonstrated a significant reduction in hospital admission and hospitalization rates. This discrepancy from the literature may be attributed to factors such as the potential for LSG to cause dyspeptic complaints, patients feeling more secure under pharmacological treatment, and overall improvements in their health status.

The findings from the literature, which suggest that UDCA can have an effect on certain liver enzymes, particularly in cholestatic diseases, but does not significantly alter all liver function parameters, are consistent with our observations. The literature indicates that UDCA results in minimal improvements in enzymes such as ALT, AST, and ALP. However, the extent of this effect may vary depending on the patient group, disease type, and treatment duration. UDCA typically does not result in direct alterations to INR or albumin levels. However, with prolonged administration, it may facilitate overall liver health and indirectly stabilize these parameters [27]. Based on these observations, it can be concluded that UDCA treatment is generally safe with regard to liver function and adverse effects, although it may induce mild gastrointestinal symptoms in some patients.

The adverse effects of UDCA are typically mild and infrequent. The most commonly reported adverse effects include diarrhea, nausea, and abdominal discomfort. In a study by Lindor et al. [28] it was reported that between 5 and 10% of patients undergoing UDCA treatment experienced mild gastrointestinal adverse effects. Moreover, although infrequent, cholestatic symptoms such as pruritus may manifest with prolonged administration. To enhance patient adherence to treatment, it is essential to strengthen doctor-patient communication, educate patients about the treatment process, and raise awareness about potential side effects.

Furthermore, the mild nature of UDCA’s side effects may be considered an advantage in promoting patient compliance. In our study, mild gastrointestinal symptoms were observed in some patients; however, these symptoms were not severe enough to hinder continued treatment. Therefore, regular follow-up, patient motivation, and monitoring of side effects are crucial for improving adherence, as adherence to treatment is a key factor that directly impacts treatment success.

### Limitations

This study is subject to several limitations. Firstly, as a retrospective design was employed, the data was collected retrospectively, which may introduce bias in patient selection and data accuracy. Secondly, the use of ultrasonography to assess stone dissolution may require more precise methods to validate stone sizes and dissolution rates. The employment of advanced imaging techniques, such as magnetic resonance cholangiopancreatography (MRCP), could provide more accurate measurements of treatment efficacy.

A further limitation of the study is that it was conducted at a single centre. The results would be more generalisable if data were obtained from different geographic regions and patient groups with diverse demographic characteristics.

## Conclusion

The results of this study demonstrate the efficacy of UDCA therapy in dissolving cholesterol gallstones. UDCA may be an effective treatment for patients who are not suitable for surgery or who decline surgical intervention. In cases of larger stones, surgical intervention may be more effective, and UDCA should primarily be considered for patients seeking non-surgical alternatives. Additionally, for larger stones, extending the pharmacological treatment duration or exploring combination therapies could offer alternative approaches.

## Data Availability

No datasets were generated or analysed during the current study.
